# Attitudes Toward Common Data Models Among Chinese Biomedical Professionals: Cross-Sectional Survey

**DOI:** 10.2196/77603

**Published:** 2025-11-05

**Authors:** Yexian Yu, Yongqi Zheng, Meng Zhang, Junqing Xie, Seng Chan You, Mengling Feng, Siyan Zhan, Feng Sun

**Affiliations:** 1 School of Information and Communication Engineering, Hainan University Haikou China; 2 Department of Epidemiology and Biostatistics, School of Public Health, Peking University Beijing China; 3 Hainan Boao Lecheng International Medical Tourism Pilot Zone Administration, Hainan Lecheng Institute of Real-World Research Lecheng China; 4 Key Laboratory of Epidemiology of Major Diseases (Peking University), Ministry of Education Beijing China; 5 Center for Statistics in Medicine NDORMS, The Botnar Research Centre University of Oxford Oxford United Kingdom; 6 Department of Biomedical Systems Informatics College of Medicine Yonsei University Seoul Republic of Korea; 7 Institute for Innovation in Digital Healthcare Severance Hospital Seoul Republic of Korea; 8 Saw Swee Hock School of Public Health National University Health System National University of Singapore Singapore Singapore; 9 Department of Ophthalmology, Peking University Third Hospital Beijing China; 10 School of Traditional Chinese Medicine, Xinjiang Medical University Urumqi China; 11 School of Public Health, Shihezi University Shihezi China

**Keywords:** attitude of health personnel, big data, common data models, electronic health records, health information exchange, medical informatics, surveys and questionnaires

## Abstract

**Background:**

In the rapidly evolving landscape of health informatics, adopting a standardized common data model (CDM) is a pivotal strategy for harmonizing data from diverse sources within a cohesive framework. Transitioning regional databases to a CDM is important because it facilitates integration and analysis of vast and varied health datasets. This is particularly relevant in China, where unique demographic and epidemiologic profiles present a rich yet complex data landscape. The significance of this research from the perspective of the Chinese population lies in its potential to bridge gaps among disparate data sources, enabling more comprehensive insights into health trends and outcomes.

**Objective:**

This study aimed to understand biomedical professionals’ and trainees’ acceptance of the CDM in medical data management in China and to explore potential advantages and challenges associated with its promotion, implementation, and development in the country.

**Methods:**

We conducted a questionnaire survey using Sojump and distributed it on WeChat to evaluate the Chinese population’s acceptance of transitioning from local databases to a standardized CDM. The survey assessed participants’ understanding of the CDM and the Observational Medical Outcomes Partnership CDM, as well as their views on the importance of CDM for regional databases in China. Analysis of the survey results revealed the current state, challenges, and trends in CDM application within Chinese health care, providing a foundation for future efforts in data standardization and sharing. The reliability of the questionnaire data was assessed using Cronbach α and Guttman Lambda 6 to determine internal consistency.

**Results:**

Our survey of 418 participants revealed that 41.9% (175/418) were aware of the CDM. Recognition of CDM increased with higher education levels and was notably higher among professionals in contract research organizations and the pharmaceutical industry. Knowledge of CDM was primarily gained through literature and conferences, with formal education less common. Logistic regression analysis indicated that individuals with doctoral degrees, researchers, executives, medical professionals, data engineers, Centers for Disease Control and Prevention staff, and statisticians were more likely to be aware of CDM. Subgroup analyses showed higher awareness among doctoral versus nondoctoral and Beijing-based versus non-Beijing respondents, while perceived necessity was broadly comparable across subgroups. Overall, 94.7% (396/418) of respondents believed CDM integration in China is necessary for standardization and efficiency. Despite 60.7% (254/418) optimism for the Observational Medical Outcomes Partnership as the preferred CDM, challenges such as mapping traditional Chinese medicine or Chinese medical insurance remain.

**Conclusions:**

A large proportion of respondents expressed a favorable view of implementing the CDM in regional databases in China, with notable endorsement from the doctoral group and professionals in contract research organizations or pharmaceutical sectors; subgroup differences were concentrated in awareness rather than perceived necessity. Participants suggested enhancing CDM-related education and establishing clear data-sharing regulations to support CDM advancement in China.

## Introduction

In the rapidly evolving landscape of health informatics, adopting a standardized common data model (CDM) is a pivotal strategy for harmonizing data from diverse sources within a cohesive framework. This approach enhances the interoperability and utility of health data and supports the advancement of precision medicine, public health monitoring, and evidence-based policy making while maintaining governance of sensitive data [1].

The transition of regional databases to a standardized CDM is vital because it facilitates the integration and analysis of large and varied health datasets. This is particularly relevant in China, where unique demographic and epidemiological profiles present a rich yet complex data landscape [2]. The significance of this research from a Chinese population perspective lies in its potential to bridge gaps between disparate data sources, enabling more comprehensive and nuanced insights into health trends and outcomes [3]. The country’s unique mix of Western medicine and Traditional Chinese Medicine, along with evolving insurance codes and inconsistent digitalization, creates challenges for data harmonization. While global studies highlight the benefits of CDMs, research on Chinese biomedical professionals’ perceptions of their adoption is limited. Globally, CDMs such as Observational Medical Outcomes Partnership (OMOP) and Vaccine Safety Datalink (VSD) have played a key role in pharmacoepidemiology, enabling large-scale, multicenter research. The United States has been a leader in using CDMs for vaccine safety and drug surveillance. These efforts offer valuable insights for China’s ongoing adoption and expansion of CDM applications [4]. Given that the CDM use in China is slightly lower than in other countries [5], a survey questionnaire was developed to investigate the perspectives and sentiments of individuals within the Chinese health care system regarding the implementation of a CDM in regional databases. Furthermore, by analyzing the factors that influence acceptance, this study aims to provide a solid foundation for promoting and expanding CDM in China’s medical field, thereby enhancing the capacity for data-driven decision-making and research.

This survey aims to examine the perspectives and sentiments of individuals within the Chinese health care industry regarding the implementation of a CDM in regional databases.

## Methods

### Ethical Considerations

The researchers explained the purpose of the study to all participants, either orally or in writing, and obtained informed consent. No compensation or incentives were provided for participation. The study was approved by the Peking University Institutional Review Board (IRB00001052-24052). No personally identifying data were collected.

### Questionnaire Survey

A questionnaire study was conducted among the Chinese population working or studying in the medical field from September 18, 2024, to November 8, 2024, to assess the acceptance of transitioning from regional databases to standardized CDMs among Chinese health care professionals. The study explored participants’ understanding of CDM and the OMOP CDM, as well as their perspectives on the importance of CDM for regional databases in China. Analysis of the survey results can reveal the current status, challenges, and trends in CDM application within the Chinese medical field, providing a reference basis for future data standardization and sharing. Survey data were collected using Sojump [6], an online survey platform, and the questionnaire was distributed through WeChat (Tencent). Reliability of the questionnaire data was assessed using Cronbach α and Guttman Lambda 6 to measure internal consistency [7,8]. The 20-question questionnaire is included in Multimedia Appendix 1. Participants viewed an information page and provided electronic informed consent before accessing the questionnaire. This web-based survey adhered to the CHERRIES (Checklist for Reporting Results of Internet E-Surveys) checklist. The fully completed checklist is available in Multimedia Appendix 2 [9].

### Statistical Analysis

The sample size was calculated to estimate the overall acceptance rate of the CDM among Chinese health care professionals. The sample size calculation used the standard proportion formula [10]. We adopted the most conservative assumption (*P*=.5) with a 95% CI (*z*=1.96) and a margin of error of 5% (E=0.05). To further justify the sample size, we reviewed similar questionnaire-based studies in the field, which reported sample sizes ranging from 249 to 683 participants [11-13]. Given this range, a sample size of 418 participants is considered appropriate and aligns with similar research.



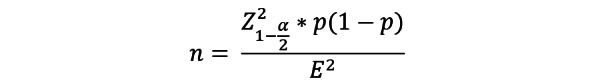



To ensure that all questions were answered before submission, the survey was designed to prevent incomplete responses, eliminating any missing data. Convenience sampling was used to efficiently gather participants for the survey. The distribution of categorical variables is expressed as numbers and percentages. Chi-square tests were used to compare nonordered categorical data. A *P* value of less than .05 was considered statistically significant. All statistical analyses were conducted using R version 4.4.1 (R Core Team).

## Results

### Basic Information

A total of 418 responses were collected. Guttman Lambda 6 reliability coefficient was 0.98, while Cronbach α coefficient was 0.96, indicating high internal consistency and strong inter-item correlations within the questionnaire. These results support the notion that the questionnaire is an effective tool, bolstering confidence in its structural soundness and reliability.

The minimum sample size was determined through a power calculation for estimating CDM acceptance prevalence among Chinese medical practitioners. Assuming a conservative proportion (*P*=50%) with a 5% margin of error at a 95% CI, the required sample size was 385. Our final sample (n=418) exceeded this threshold, ensuring sufficient precision for population-level estimates. Required sample sizes across various assumed proportions are shown in Table 1.

**Figure 1:**
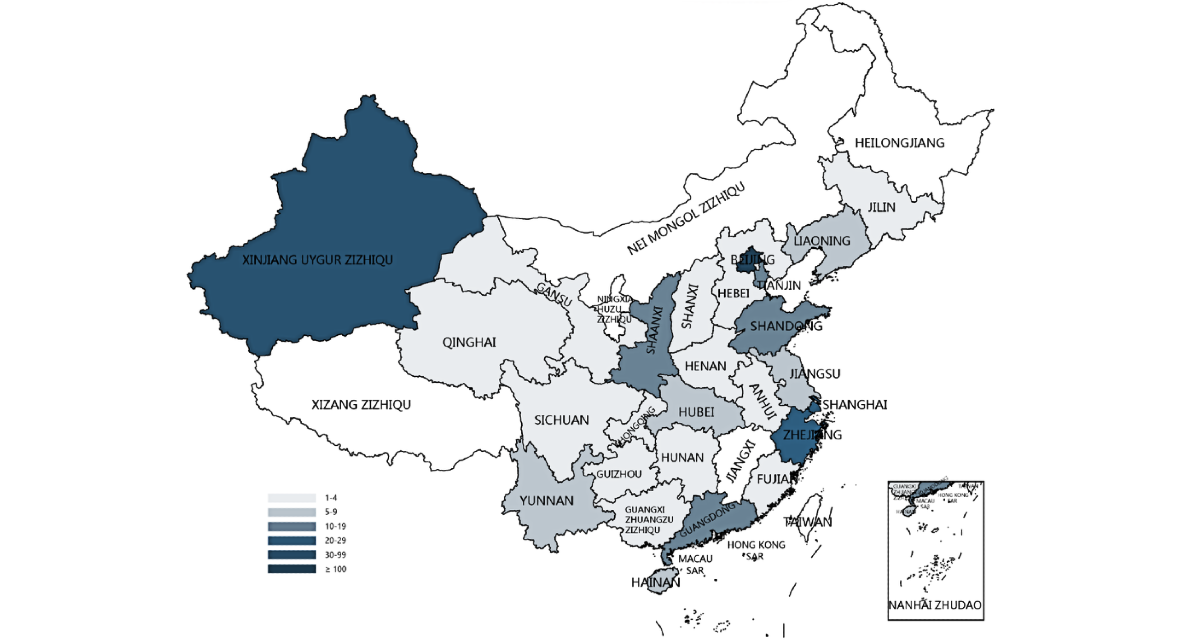
Regional distribution of questionnaire filling in CHINA.

The median filling time was 121.5 (IQR 83.0-201.5) seconds. Zhong et al [14] recommend estimating the time required to answer a question as approximately 2 seconds per question, which all participants met in this study. Among the participants, 40.2% (168/418) were from Beijing and were notably more engaged, indicating a higher level of interest in the topic. This was followed by Xinjiang at 20% (84/418). Furthermore, significant interest was also observed in economically prosperous regions such as Zhejiang (21/418, 5%), Shanghai (20/418, 5%), and Guangdong (16/418, 4%). This may be related to economic development, medical resource allocation, and enhanced health awareness among residents in these areas. The regional distribution of participants is shown in Figure 1 [15], and participant characteristics are shown in Table 2.

**Table 1:** Sample size calculation.

Anticipated acceptance rate (p)	Required sample, n
10% or 90%	138
20% or 80%	246
30% or 70%	323
40% or 60%	369
50%	385

**Table 2:** Participant characteristics.

Factors			Frequency, n (%)
	Male	255 (61)	
	Female	163 (39)	
**Age group (years)**			
	18-30	104 (24.88)	
	31-40	165 (39.47)	
	41-50	115 (27.51)	
	51-60	27 (6.46)	
	>60	7 (1.67)	
**Educational attainment**			
	Bachelor’s	122 (29.19)	
	Master’s	191 (45.69)	
	Doctor	105 (25.12)	
**Professional roles**			
	Students	55 (13.16)	
	Researchers	101 (24.16)	
	Executives	55 (13.16)	
	Medical professionals	109 (26.08)	
	Teachers	22 (5.26)	
	Data engineers	14 (3.35)	
	CDC^a^ staff	19 (4.55)	
	Statisticians	18 (4.31)	
	Others^b^	25 (5.98)	
**Institutional Affiliation**			
	CDC	143 (34.21)	
	Pharmaceutical industries	46 (11)	
	Colleges	80 (19.14)	
	Hospital	63 (15.07)	
	CROs^c^	41 (9.81)	
	Others^d^	45 (10.77)	

^a^CDC: Centers for Disease Control and Prevention.

^b^The others mentioned in professional roles refer to sales, administration, and not specified.

^c^CRO: contract research organization.

^d^The others mentioned in institutional affiliation refer to research institutes, the National Health Commission of China, and not specified.

### Degree of CDM Understanding

A total of 41.9% (175/418) of participants had heard of CDM before, whereas in the doctoral group, the percentage was higher at 60% (63/105). There was a statistically significant association between awareness of CDM and education level (*χ*²_2_=22.2; *P*<.001), occupation (*χ*²_8_=33.1; *P*<.001), and working or studying area (*χ*²_5_=46.1; *P*<.001), while no significant differences were observed in terms of age group and sex.

**Figure 2:**
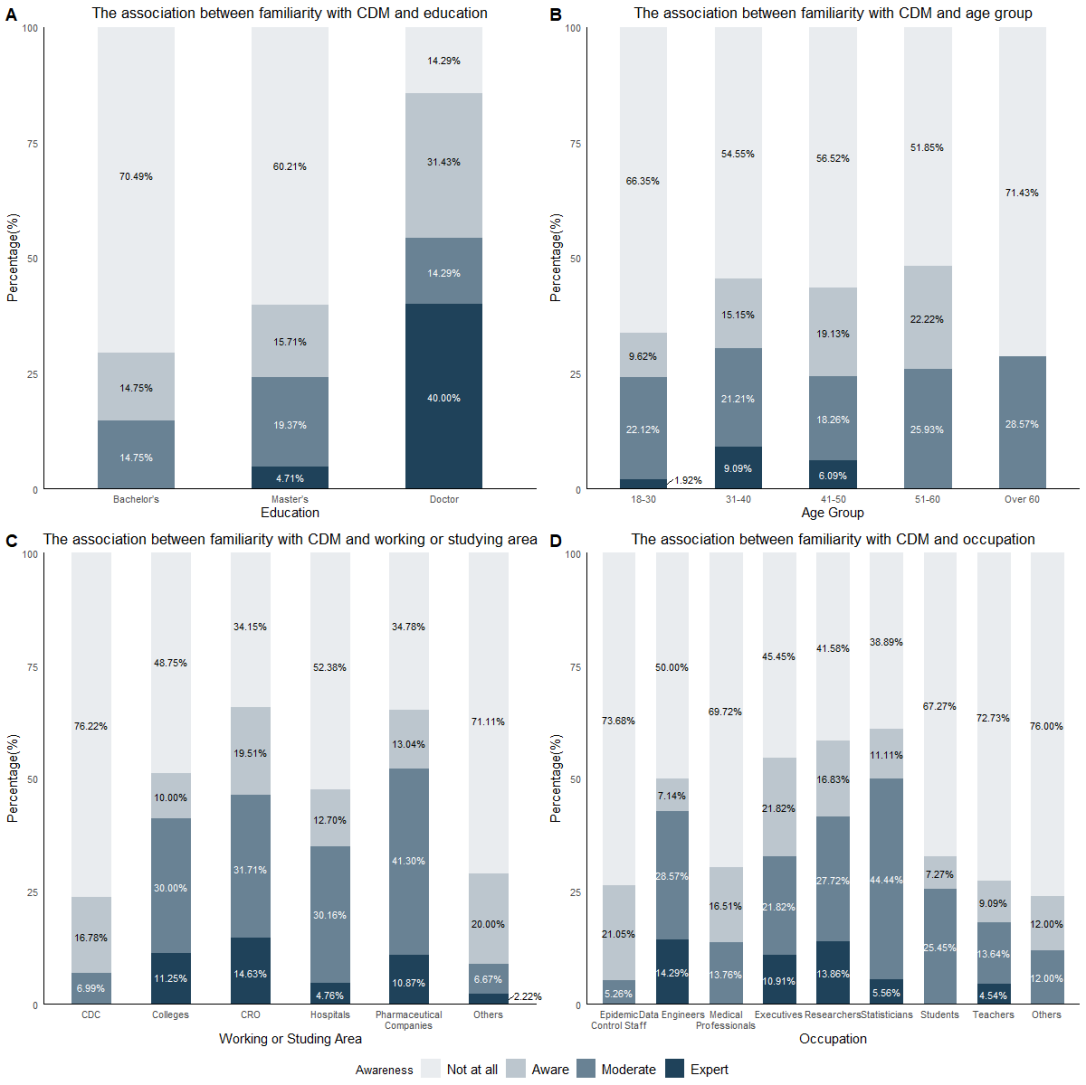
The association between familiarity with CDM and different factors. CDC: Centers for Disease Control and Prevention; CDM: common data model; CRO: contract research organization.

Among participants who were aware of CDM, only 14% (24/175) reported having a comprehensive understanding, whereas within the doctoral population, this percentage increased to 24% (15/63). Figure 2 illustrates a positive correlation between education level and the extent of CDM understanding. Notably, familiarity with CDM tends to decrease with increasing age. Specifically, within the 31-40 years age group, 9% (15/165) of participants demonstrated a profound understanding of CDM, representing the highest proportion among all age groups. Contract Research Organizations (CROs) and the pharmaceutical industry exhibit heightened interest in CDM, potentially attributable to the elevated demand for scientific research within these sectors. Data engineers and researchers typically reported higher familiarity with CDMs, and executives also reported relatively high levels of understanding.

Among the 175 participants familiar with CDMs, conferences and academic literature emerged as the 2 primary sources of knowledge, cited by 119 (68%) and 109 (62.3%) respondents, respectively. Digital platforms and informal networks also played notable roles: medical subscription accounts, such as medical public accounts or newsletters on WeChat and similar platforms, were mentioned by 64 respondents (37%), information shared by peers by 58 (33%), and social media by 31 (18%). In contrast, formal education served as a source for only 3 participants (2%), highlighting a considerable gap in structured curricula and suggesting an urgent need to integrate CDM training into established academic and professional education programs.

Univariate and multivariate logistic regression analyses were conducted to assess the association between having heard of CDM and various demographic and clinical characteristics. The results are presented in Table 3. No differences between univariate and multivariate models were observed for age groups. Regarding education level, individuals with a doctoral degree (odds ratio [OR] 2.751, 95% CI 1.214-6.345; *P*=.02) exhibited a significantly greater likelihood of having heard of CDM compared with those with an undergraduate degree, whereas no significant difference was observed for those holding a master’s degree. Researchers (OR 5.893, 95% CI 1.520-9.469; *P*=.02), executives (OR 5.716, 95% CI 1.179-9.844; *P*=.04), medical professionals (OR 4.964, 95% CI 1.984-8.162; *P*=.008), data engineers (OR 1.782, 95% CI 1.009-7.707; *P*=.01), Centers for Disease Control and Prevention (CDC) staff (OR 2.139, 95% CI 1.519-6.809; *P*=.02), and statisticians (OR 3.746, 95% CI 1.336-6.878; *P*=.03) all had previous knowledge of CDM compared with students. This suggests that the concept of CDM has gained some recognition within their professional fields. Such preexisting awareness may stem from their educational background, work experience, or attention to industry trends. It is noteworthy that this early familiarity with CDM could have influenced their attitudes and behaviors toward participation in the study and may also reflect their professional competence in data management. Specifically, compared with those working or studying at the CDC, individuals in the pharmaceutical industry (OR 3.993, 95% CI 2.691-6.940; *P*<.001), CROs (OR 3.138, 95% CI 1.289-8.409; *P*<.001), and colleges (OR 4.650, 95% CI 1.680-8.335; *P*<.001) demonstrated greater awareness of CDM.

**Table 3:** Univariate and multivariate logistic regression analysis for common data models awareness.

Factors			Univariate						Multivariate				
			OR^a^	95% CI		*P* value		OR			95% CI		*P* value
**Age group (years)**													
	18-30	Reference		—^b^	—		Reference			—		—	
	31-40	1.643		0.992-2.751	.06		1.217			0.534-2.884		.65	
	41-50	1.516		0.878-2.638	.14		1.284			0.537-3.187		.58	
	51-60	1.831		0.771-4.339	.17		1.967			0.581-6.748		.28	
	>60	0.789		0.109-3.864	.78		1.468			0.513-9.884		.98	
**Education attainment**													
	Bachelor’s	Reference		—	—		Reference			—		—	
	Master’s	1.579		0.977-2.581	.07		1.210			0.667-2.202		.53	
	Doctor	3.583		2.079-6.272	<.001		2.751			1.214-6.345		.02	
**Professional roles**													
	Students	Reference		—	—		Reference			—		—	
	Researchers	2.887		1.467-5.842	.003		5.893			1.520-9.469		.02	
	Executives	0.467		1.148-5.426	.02		5.716			1.179-9.844		.04	
	Medical professionals	0.893		0.447-1.810	.75		4.964			1.984-8.162		.008	
	Teachers	0.771		0.243-2.232	.64		0.398			0.096-15.113		.19	
	Data engineers	2.056		0.617-6.897	.24		1.782			1.009-7.707		.01	
	CDC^c^ staff	0.734		0.211-2.260	.60		2.139			1.519-6.809		.02	
	Statisticians	3.230		1.092-10.147	.04		3.746			1.336-6.878		.03	
	Others^d^	0.649		0.207-1.840	.37		2.761			0.456-18.915		.28	
**Institutional affiliation**													
	CDC	Reference		—	—		Reference			—		—	
	Pharmaceutical industries	6.011		2.971-12.585	<.001		3.993			2.691-6.940		<.001	
	Colleges	3.370		1.890-6.085	<.001		4.650			1.680-8.335		<.001	
	Hospitals	3.282		1.564-7.003	.002		2.914			1.560-5.483		<.001	
	CROs^e^	6.183		2.963-13.425	<.001		3.138			1.289-8.409		<.001	
	Others^f^	1.302		0.600-2.722	.49		2.036			0.787-5.299		.14	

^a^OR: odds ratio.

^b^Not applicable.

^c^CDC: Centers for Disease Control and Prevention.

^d^Others refer to sales, administration, and not specified.

^e^CRO: contract research organization.

^f^Others refer to research institutes, the National Health Commission of China, and not specified.

### Necessity of CDM

Considering that some participants were not familiar with CDM and had only a vague understanding of its definition, the definition of CDM was provided in the questionnaire. After the definition was clarified on the first page, 94.7% (396/418) of participants indicated that incorporating CDM into regional databases in China is necessary. Univariate and multivariate logistic regression analyses were conducted to assess perceptions of CDM necessity, and the results are presented in Table 4. In univariate analysis, individuals in the 31-40 years and 41-50 years age groups were more likely to be aware of CDM as necessary compared with the 18-30 years age group, whereas individuals aged 51-60 years and older showed the opposite tendency. In multivariate regression analysis, after adjusting for confounders, the differences between the 31-40 years and 41-50 years age groups and the 18-30 years age group were no longer statistically significant, whereas individuals in the 51-60 years (OR 0.205, 95% CI, 0.065-0.602; *P*=.005) and 60 years and older (OR 0.050, 95% CI 0.019-0.313; *P*=.003) age groups still held significantly different attitudes. Individuals with master’s (OR 2.153, 95% CI 1.054-4.516; *P*=.04) and doctoral (OR 3.083, 95% CI 1.317-7.470; *P*=.01) degrees showed a higher degree of agreement with CDM as necessary than those with bachelor’s degrees, which remained significant after controlling for other potentially confounding variables, suggesting an independent association between education level and perception of the need for CDM. Researchers (OR 2.829, 95% CI 1.301-5.808; *P*=.03), executives (OR 4.227, 95% CI 1.198-7.236; *P*=.03), medical professionals (OR 4.189, 95% CI 1.279-8.196; *P*=.02), data engineers (OR 5.014, 95% CI 1.593-9.558; *P*=.04), and CDC staff (OR 3.371, 95% CI 1.098-5.315; *P*=.008) all agreed that CDM is necessary compared with students. Considerable differences were observed in awareness and perceived requirements for CDM among different work and study environments. Specifically, staff in pharmaceutical industries (OR 2.107, 95% CI 1.846-8.139; *P*=.003) were more likely to perceive CDM as necessary than CDC staff, while those in CROs (OR 0.340, 95% CI 0.135-0.941; *P*=.04) showed the opposite view. In addition, it is important to note that staff in other areas (OR 4.701; 95% CI 1.453-9.624; *P*=.02), such as research institutes and the National Health Commission, were more likely than CDC staff to agree with the need for CDM. This phenomenon may reflect different understandings and levels of awareness of the value of CDM and the urgency of its implementation in various areas.

**Table 4:** Univariate and multivariate logistic regression analysis for common data models necessity.

Factors		Univariate							Multivariate				
		ORᵃ		95% CI		*P* value		OR			95% CI		*P* value
**Age group (years)**													
	18-30	Reference	—ᵇ		—		Reference			—		—	
	31-40	2.378	1.281-4.497		.007		0.981			0.371-2.508		.97	
	41-50	2.101	1.075-4.228		.03		0.934			0.333-2.571		.90	
	51-60	0.294	0.145-0.582		.001		0.205			0.065-0.602		.005	
	>60	0.225	0.075-0.596		.004		0.050			0.019-0.313		.003	
**Education attainment**													
	Bachelor’s	Reference	—		—		Reference			—		—	
	Master’s	2.912	1.739-4.943		<.001		2.153			1.054-4.516		.04	
	Doctor	1.676	1.295-2.917		.006		3.083			1.317-7.470		.01	
**Professional roles**													
	Students	Reference	—		—		Reference			—		—	
	Researchers	1.851	1.068-3.576		.005		2.829			1.301-5.808		.03	
	Executives	1.270	2.744-5.916		.54		4.227			1.198-7.236		.03	
	Medical professionals	1.920	1.285-3.773		.006		4.189			1.279-8.196		.02	
	Teachers	1.669	0.285-3.654		.98		1.205			0.529-5.621		.99	
	Data engineers	4.267	0.974-9.315		.08		5.014			1.593-9.558		.04	
	CDCᶜ staff	6.044	1.806-9.781		.008		3.371			1.098-5.315		.008	
	Statisticians	1.669	0.795-3.645		.99		1.538			0.085-9.548		.99	
	Othersᵈ	0.960	0.338-2.701		.94		0.307			0.056-1.571		.16	
**Institutional affiliation**													
	CDC	Reference	—		—		Reference			—		—	
	Pharmaceutical industries	5.667	1.529-8.412		.02		2.107			1.846-8.139		.003	
	Colleges	0.608	0.332-1.109		.11		0.698			0.255-1.907		.48	
	Hospitals	0.886	0.494-1.592		.69		0.837			0.373-1.905		.67	
	CROsᵉ	0.349	0.159-0.750		.007		0.340			0.135-0.941		.04	
	Othersᶠ	1.495	0.612-4.053		.40		4.701			1.453-9.624		.02	

^a^OR: odds ratio.

^b^Not applicable.

^c^CDC: Centers for Disease Control and Prevention.

^d^Others refer to sales, administration, and not specified.

^e^CRO: contract research organization.

^f^Others refer to research institutes, the National Health Commission of China, and not specified. The majority of respondents, 93.7% (371/396), identified the integration of heterogeneous health big data from multiple sources and the enhancement of data consistency as key rationales for the implementation of CDM, underscoring the significance of data integration and consistency in contemporary data management practices. In addition, 87.1% (345/396) of respondents highlighted the crucial role of structured data in augmenting the efficiency of scientific research endeavors, whereas 81.8% (324/396) of respondents emphasized the importance of improving data quality and reliability. Furthermore, 69.4% (275/396) of respondents, representing a substantial proportion of the sample, regarded the enhancement of data use rates and the conservation of storage space as another pivotal incentive for the adoption of CDM. Collectively, these data reveal the multidimensional value of CDM in facilitating data standardization, optimizing research processes, bolstering data reliability, and enhancing storage efficiency. In contrast, 5% (22/418) participants believed that CDM is not necessary for regional databases in China. More than half (12/22, 55%) felt that existing databases already meet their needs. A total of 45% (10/22) of respondents indicated that the implementation and maintenance of CDM are relatively complex and costly, whereas 36% (8/22) expressed concerns regarding data security and the potential leakage of patient privacy.

### CDM Type

Among the databases known to participants, the top 5 included OMOP, VSD, Sentinel, Clinical Data Acquisition Standards Harmonization, and National Patient-Centered Clinical Research Network, with OMOP having a significantly higher awareness rate (Table 5). It is particularly noteworthy that while only 16% (21/132) of respondents working at the CDC chose OMOP, this figure increases to 24% (11/46) among those who selected VSD, possibly because their work provides them with a better understanding of VSD in the vaccine field [16]. Within this subgroup of 175 respondents who had heard of CDMs, only 14 (8%) individuals reported knowledge of more than 5 types. By contrast, when considering the entire sample of 418 participants, as many as 327 (78.2%) indicated that they were unfamiliar with any type of CDM. In recent years, Chinese medical institutions have progressively explored the application of OMOP CDM in real-world studies. As demonstrated in a systematic review conducted in 2023 [17], 12 institutions nationwide had successfully implemented data standardization, with notable breakthroughs particularly in chronic and mental disease research.

**Table 5:** Ranking of common data model (CDM) types known to participants.

Ranking	CDM type	Participants, n/N (%)
1	OMOP^a^	132/175 (75.4)
2	VSD^b^	45/175 (26)
3	Sentinel	41/175 (23)
4	CDASH^c^	36/175 (21)
5	PCORnet^d^	28/175 (16)
6 (tie)	ASPEN^e^	23/175 (13)
6 (tie)	FHIR^f^	23/175 (13)
8 (tie)	i2b2^g^	21/175 (12)
8 (tie)	PEDSnet^h^	21/175 (12)
10	CRN-VDW^i^	20/175 (11)
11	ConcePTION	10/175 (6)

^a^OMOP: Observational Medical Outcomes Partnership.

^b^VSD: Vaccine Safety Datalink.

^c^CDASH: Clinical Data Acquisition Standards Harmonization.

^d^PCORnet: National Patient-Centered Clinical Research Network.

^e^ASPEN: Advanced System for Process Engineering.

^f^FHIR: Fast Health Interoperability Resources.

^g^i2b2: Informatics for Integrating Biology & the Bedside.

^h^PEDSnet: Pediatric Evidence Discovery and Surveillance Network.

^i^CRN-VDW: Health Care Systems Research Network Virtual Data Warehouse.

Equal counts were assigned the same rank (ties); subsequent ranks were skipped.

**Figure 3:**
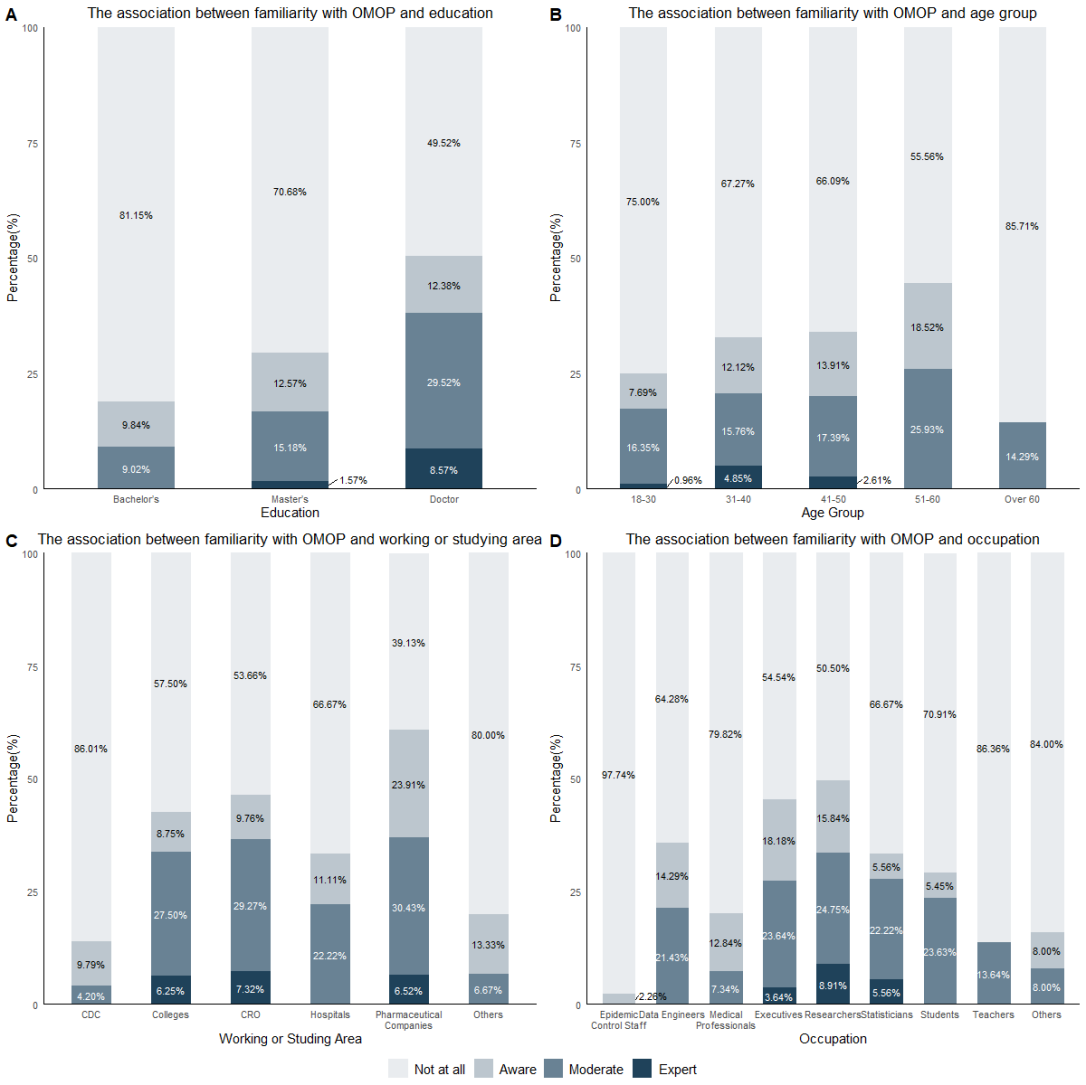
The association between familiarity with OMOP and different factors. CDC: Centers for Disease Control and Prevention; CRO: contract research organization; OMOP: Observational Medical Outcomes Partnership.

The bar charts illustrating familiarity with OMOP (Figure 3) appear to closely resemble those for CDM. Among the 132 respondents who reported being aware of OMOP, the primary avenues for OMOP awareness were conference introductions and literature, reported by 90 (68%) respondents and 75 (57%) respondents, respectively, underscoring the significant role of professional events and scholarly resources in knowledge dissemination. In addition, 44 participants (33%) learned about OMOP from peers, and 41 (31%) from medical public accounts, while only 13 respondents (10%) obtained such information through video platforms. Survey findings indicate that the majority of respondents recognize significant advantages associated with the implementation of OMOP. The most widely acknowledged benefit, reported by 89.4% (118/132) of participants, is that it enables different data sources to be more easily shared, compared, and integrated. This is followed by the enhancement of data operability and comparability, cited by 104 respondents (78.8%). Furthermore, 98 respondents (74%) believe that OMOP helps promote innovation and progress within the research field. Additionally, a substantial proportion of respondents, 64% (84/132), identified the reduction in data cleaning workload as a notable advantage. However, respondents also identified challenges faced by OMOP in China. Specifically, 76% (100/132) of respondents aware of OMOP cited barriers between various data sources, 71% (94/132) reported the high manual effort required for mapping, and 52% (69/132) encountered an inability to match some localized information to standard concept IDs. Particular mapping issues were noted for data related to ethnicity, Chinese medical insurance, and traditional Chinese medicine.

### Subgroup Analysis

To examine whether sample composition might influence the main results and to enhance interpretability, we conducted prespecified subgroup comparisons by education (doctoral vs nondoctoral) and region (Beijing-based vs non–Beijing-based) in Tables 6 and 7, respectively. Doctoral respondents showed higher CDM awareness (*χ*²_1_=11.1; *P*<.001), greater self-rated CDM expertise (*χ*²_1_=16.9; *P*<.001), and higher OMOP awareness (*χ*²_1_=22.0; *P*<.001). Perceived necessity did not differ materially by education (*P*=.31 for CDM and *P*=.15 for OMOP). By region, Beijing-based participants had higher awareness of CDM (*χ*²_1_=5.1; *P*=.02) and OMOP (*χ*²_1_=14.2; *P*<.001), whereas differences in CDM and OMOP expertise and perceived necessity were not statistically significant. Overall, heterogeneity was concentrated in awareness rather than perceived necessity, suggesting differential exposure rather than divergent attitudes toward adoption.

**Table 6:** Subgroup analysis by education level. Statistical methods: Pearson χ² test was used when all expected cell counts were >5; otherwise, the Fisher exact test was applied.

Items		Doctoral degree (N=105), n (%)	Nondoctoral degree (N=313), n (%)	Chi-square (*df*)		*P* value	
**CDM^a^ awareness**					11.1 (1)		<.001
	Yes	63 (60)	139 (40.88)				
	No	42 (40)	201 (59.12)				
**CDM expertise**					16.9 (1)		<.001
	Yes	15 (14.29)	9 (2.88)				
	No	90 (85.71)	304 (97.12)				
**CDM necessity**					—^b^		.31
	Yes	102 (97.14)	297 (93.99)				
	No	3 (2.86)	19 (6.01)				
**OMOP** ^c^ **awareness**					22.0 (1)		<.001
	Yes	53 (50.48)	79 (25.24)				
	No	52 (49.52)	234 (74.76)				
**OMOP expertise**					—		<.001
	Yes	9 (8.57)	3 (0.96)				
	No	96 (91.43)	310 (99.04)				
**OMOP necessity**					2.1 (1)		.15
	Yes	57 (54.29)	197 (62.94)				
	No	48 (45.71)	116 (37.06)				

^a^CDM: common data model.

^b^Not available.

^c^OMOP: Observational Medical Outcomes Partnership.

**Table 7:** Subgroup analysis by geographic region. Statistical methods: Pearson χ² test was used when all expected cell counts were >5; otherwise, the Fisher exact test was applied.

Items			Beijing-based (N=168), n (%)		Non-Beijing–based (N=250), n (%)		Chi-square (*df*)		*P* value
**CDM^a^ awareness**							5.1 (1)		.02
	Yes	82 (48.81)		93 (37.20)					
	No	86 (51.19)		157 (62.80)					
**CDM expertise**							0.6 (1)		.43
	Yes	12 (7.14)		12 (4.80)					
	No	156 (92.86)		238 (95.20)					
**CDM necessity**							1.4 (1)		.24
	Yes	156 (92.86)		240 (96)					
	No	12 (7.14)		10 (4)					
**OMOP** ^b^ **awareness**							14.2 (1)		<.001
	Yes	60 (35.71)		47 (18.80)					
	No	108 (64.29)		203 (81.20)					
**OMOP expertise**							—^c^		.99
	Yes	5 (2.98)		7 (2.80)					
	No	163 (97.02)		243 (97.20)					
**OMOP necessity**							2.4 (1)		.12
	Yes	94 (55.95)		160 (64)					
	No	74 (44.05)		90 (36)					

^a^CDM: common data model.

^b^OMOP: Observational Medical Outcomes Partnership.

^c^Not available.

## Discussion

Based on the statistical analysis of the questionnaire results from Sojump, it is evident that participants generally hold a positive attitude toward the application of CDM in regional databases in China. This positivity is underpinned by the high internal consistency and strong inter-item correlations within the questionnaire. Notably, participants from Beijing constituted the highest proportion, which may indicate a regional focus or interest in CDM. The doctoral group, with their deeper professional knowledge and experience in the medical and research fields, tended to have a higher understanding of CDM. This understanding equips them to comprehend the structure and potential applications of CDM, and how to use it to support clinical research and health care decision-making. Furthermore, CROs and pharmaceutical industries showed a strong interest in CDM due to its standardized data models and processing methods, which can accelerate project processes, reduce costs, and enhance efficiency. This interest and support are instrumental in driving the widespread adoption of CDM, thereby providing more opportunities for data management and analysis in the health care sector. Literature and conferences were identified as the primary channels through which participants learned about CDM. Among various CDM frameworks, OMOP ranked highest in terms of awareness. However, participants generally believed that implementing OMOP in China would face numerous challenges, such as the inability to map traditional Chinese medicine information to standard datasets. Mapping challenges in China include the difficulty of translating traditional Chinese medicine concepts, such as “Qi deficiency” and “Blood stagnation,” into OMOP-standardized terms, as no direct equivalents exist. Similarly, local insurance and billing codes, which are frequently updated and vary by region, present significant challenges in adapting them to the OMOP framework.

Some local adaptations are underway, with certain Chinese institutions developing crosswalks and custom vocabularies to address these challenges. However, these adaptations are still in the pilot phase and require further collaboration to be fully integrated into the global OMOP ecosystem.

Participants put forward a series of recommendations to enhance the involvement of domestic technical, medical, informational, and scientific research personnel and to promote the development of CDM in China. They emphasized the importance of establishing small-scale OMOP data specialty and disease-specific databases, as well as the possibility of building OMOP thematic databases based on existing databases. To increase the operational strength of OMOP data, they suggested increasing investment in scientific research and commercial projects to enhance scientific and commercial outputs. At the same time, the necessity of popularizing CDM education was highlighted, along with an emphasis on policy precedence. They hope to develop a CDM suitable for China that aligns with international standards, while also focusing on quality control in data collection. Additionally, they discussed the feasibility of using Clinical Data Interchange Standards Consortium standards in real-world data and suggested a comprehensive promotion of the use of a unified data format. Participants also stressed the importance of establishing rules to clarify the value of data assets and the need for national-level promotion of a rational data-sharing mechanism. Questions were raised regarding whether to charge fees, how to charge fees, and the possibility of customization, emphasizing the importance of data integration, collection, and processing. It was suggested to popularize CDM in universities and consider opening and sharing some data with enterprises. At the same time, the importance of information encryption and network security was emphasized, along with a proposal to pilot initiatives in specific areas first. Finally, they discussed the issue of defining cross-institutional data usage permissions and strategies to enhance users’ enthusiasm.

The recommendations put forward by the participants hold significant guiding importance in promoting the application and development of CDM and OMOP in China. Standardization and privacy are complementary facets of data sharing, where CDM can serve as an effective solution for both. Standardization facilitates and enhances the efficiency of data sharing, while privacy ensures that data sharing is conducted appropriately and under sufficient control. Recent nationwide implementations, such as South Korea’s successful integration of national claims data into the OMOP CDM framework, demonstrate how standardization can support both data utility and privacy protection at a national scale. This approach enables transparent and “Findable, Accessible, Interoperable, and Reusable” data stewardship while maintaining appropriate access controls through distributed research environments [18]. There is active development in both domains, including advancements in government regulations and common data models to promote standardization, as well as the application of technologies such as blockchain and synthetic data to address privacy concerns [19,20]. Enhancing the interoperability and security of information systems is crucial, necessitating increased awareness and implementation of current standards and the expansion of ophthalmic data standards to fill gaps in existing norms. As data sharing becomes more prevalent, advancements in data privacy are also of critical importance.

The use of OMOP has facilitated standardized data-driven research across various clinical sites, establishing a robust foundation for data use. Furthermore, numerous research papers in the medical field have used OMOP to present their findings. Artificial intelligence is increasingly being applied within CDM. Kang et al [21] introduced a deep learning–based automatic term mapping tool specifically designed for application within the OMOP framework, enhancing the efficiency and accuracy of data standardization. The review by Ahmadi et al [22] highlights the pivotal role of OMOP in discovering and refining machine learning models. Some studies have explored disease risk factors in detail and conducted epidemiological investigations [23-27], while others have used machine learning to build risk prediction models [28-32].

This study has several limitations. First, while the sample size of 418 meets basic statistical requirements, it remains relatively limited for comprehensively assessing the perspectives of all health care professionals and researchers across China’s diverse health care landscape. Second, this study used convenience sampling, which may introduce selection bias, as the selection of participants was not entirely random and may not fully reflect the opinions and characteristics of all health care professionals and researchers in China. Convenience sampling likely overrepresented highly educated professionals, particularly those with doctoral degrees, and respondents from regions such as Beijing. These biases may lead to more favorable attitudes toward CDMs and higher familiarity with them compared with the broader population of health care professionals in China. To improve the accuracy and reliability of future studies, stricter sampling techniques should be adopted to ensure sample diversity and broadness. Additionally, other potential biases in the study design and implementation process need to be addressed in subsequent research. Furthermore, the assessment of “comprehensive knowledge” of CDMs was based on self-reported responses rather than an objective knowledge test. While this method provides valuable insights into participants’ perceptions, it may not fully capture their actual understanding of CDMs. Given the online survey format, digital literacy could be a potential source of bias. However, as all respondents held at least a bachelor’s degree and were capable of using electronic devices, the impact of this bias is likely minimal.

In conclusion, respondents were generally supportive of adopting CDMs in regional databases in China, with higher awareness among doctoral respondents and staff in CROs or pharmaceutical sectors; however, deep understanding remains limited, and exposure through formal training is low. Accordingly, these findings should be interpreted with caution. Implementation efforts should prioritize targeted education and capacity-building, alongside clear data-sharing and governance frameworks, as well as practical pilots—including terminology mapping—to enable sustainable uptake.

## Appendix

Multimedia Appendix 1 Questionnaires.

Multimedia Appendix 2 CHERRIES Checklist.

## Abbreviations

CDC: Centers for Disease Control and Prevention
CDM: common data model
CHERRIES: Checklist for Reporting Results of Internet E-Surveys
CRO: contract research organization
OMOP: Observational Medical Outcomes Partnership
OR: odds ratio
VSD: Vaccine Safety Datalink

## Data Availability

The datasets generated or analyzed during this study are available in the GitHub repository [33] under a Creative Commons Attribution 4.0 International License (CC BY 4.0).
