# Supplementary material for: Attitudes Toward Common Data Models Among Chinese Biomedical Professionals: Cross-Sectional Survey
Source: JMIR Med Inform. 2025 Nov 5;13:e77603. doi: 10.2196/77603 (PMC12631093; doi:10.2196/77603)

**All of the questions in the questionnaire (Translate version)**

Dear Respondent,

Greetings!

Thank you for participating in this survey on the acceptance of transforming regional databases into a standardized Common Data Model (CDM) from the perspective of the Chinese population. Before we begin, we would like to briefly introduce the concept of Common Data Models:

No single observational database can comprehensively record all clinical events experienced by patients during their medical care. Therefore, research findings must be extracted from multiple different data sources, compared, and analyzed to understand the potential impact of recording biases. Additionally, to draw statistically significant conclusions, large-scale observational studies involving numerous patients are required, which explains the necessity of evaluating and analyzing multiple data sources simultaneously. To achieve this, diverse datasets must be governed under a unified common data standard. Furthermore, patient data must be strictly protected. Traditionally, extracting data for analysis requires strict data use agreements and complex access controls. However, adopting a common data standard eliminates the need for data extraction, enabling standardized analysis to be conducted within the local environment—where the analysis is performed at the data source rather than extracting the data first. The Common Data Model (CDM) is precisely such a universal data standard.

Based on the above explanation, we invite you to participate in this survey and share your perspectives and suggestions on the feasibility of implementing Common Data Models in China’s regional databases. Your participation is of great significance to our research. Please read the questions carefully and answer them truthfully according to your actual experience. Your responses will be used solely for academic purposes, and we will strictly protect your personal information. All data will be used only for statistical analysis and will not be utilized for any other purposes.

Thank you for your cooperation! We wish you a pleasant experience in completing the survey and look forward to your valuable insights and suggestions.

School of Public Health, Peking University

| **1. Your gender?** |
| --- |
| - Male |
| - Female |
| **2. Your age group?** |
| - Under 18 |
| - 18-30 |
| - 31-40 |
| - 41-50 |
| - 51-60 |
| - Over 61 |
| **3. Whether or not you work/study in the medical field?** |
| - Yes |
| - No |
| **4. Which of the following applies?** |
| - Hospitals |
| - Colleges |
| - CDC |
| - CRO Companies |
| - Pharmaceutical companies |
| - Others |
| **5. Your job?** |
| - Doctors or nurses |
| - Scientists |
| - Teachers |
| - Students |
| - Middle and senior management |
| - Data Engineer |
| - Others |
| **6. Your educational background?** |
| - Bachelor or less |
| - Master |
| - Ph. D |
| **7. Have you heard of CDM before?** |
| - Yes |
| - No (No.8, No.9, No.13, No.14 and No.15 were skipped) |
| **8. How well do you know CDM?** |
| - Just heard about it but don't know much about it |
| - Learn a little |
| - Very well understood |
| **9. Where did you hear about CDM?** |
| - Literature |
| - Conferences |
| - Medical public account |
| - Social media |
| - Informed by other people |
| - Others |
| **10. Do you think a CDM is necessary (after giving the definition of CDM)?** |
| - Yes (Skip to No.11) |
| - No (Skip to No.12) |
| **11. The reasons why you think CDM is necessary.** |
| - Structured data to improve the efficiency of scientific research |
| - Integrate multi-source and heterogeneous health big data to improve data consistency |
| - Improve data quality and trustworthiness |
| - Improve data utilization and save storage space |
| - Others |
| **12. The reasons why you think CDM is unnecessary.** |
| - Implementation and maintenance are complex and cost-prohibitive |
| - The existing system is sufficient |
| - Concerns about data security and patient privacy breaches |
| - Others |
| **13. What CDM do you know?** |
| - OMOP |
| - VSD |
| - i2b2 |
| - CRN-VDW |
| - PCORnet |
| - PEDSnet |
| - Sentinel |
| - ASPEN |
| - FHIR |
| - CDASH |
| - ConcePTION |
| - Others |
| **14. How well do you know OMOP CDM?** |
| - Just heard about it but don't know much about it |
| - Learn a little |
| - Very well understood |
| **15. Where did you hear about OMOP CDM?** |
| - Literature |
| - Conferences |
| - Medical public account |
| - Social media |
| - Informed by other people |
| - Others |
| **16. Do you think OMOP CDM will become the mainstream CDM choice for regional databases in China in the future? (after giving the definition of OMOP)?** |
| - Yes |
| - No |
| - I don't know |
| **17. What benefits do you think OMOP CDM can bring to regional databases in China?** |
| - Improve the operability and comparability of data - Different data sources can be easily shared, compared, and integrated - Contribute to driving innovation and progress in the field of research - Reduce the effort of data cleansing |
| - Others |
| **18. What do you think are the current challenges that OMOP CDM is facing in China?** |
| - Mapping is labor-intensive |
| - Code reproduction is complex |
| - The cost is too high |
| - Diversity of data sources |
| - There are barriers between data sources |
| - Some of the Chinese information cannot be matched to the standard Concept ID (Skip to No. 19) |
| - Others |
| **19. Have you seen what information doesn't match to a standard Concept ID?** |
| - Ethnic group |
| - Information related to medical insurance in China |
| - Information related to traditional Chinese medicine |
| **20. Do you have any other views and suggestions on the application of CDM in China's regional databases?** |

**The original Chinese version is available upon request by scanning the QR code below.**


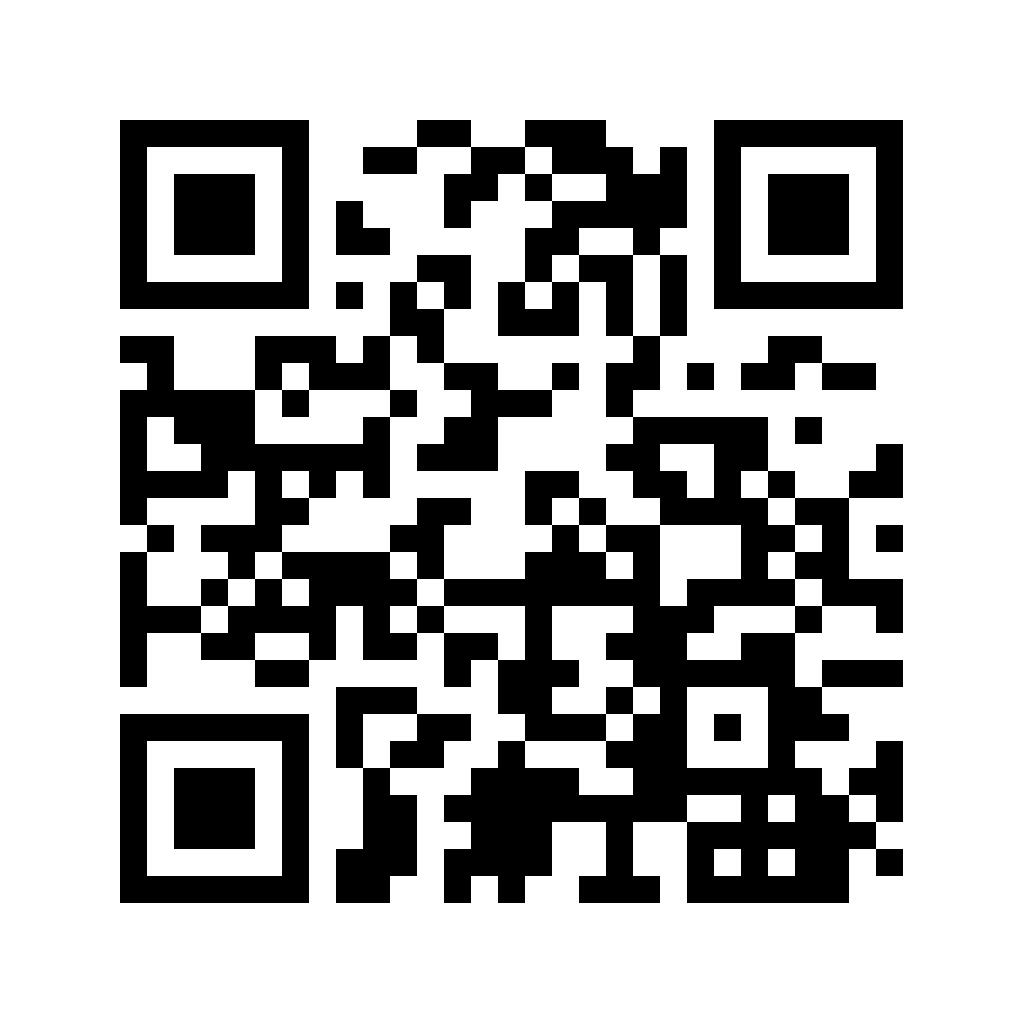

Supplement: Multimedia Appendix 1 [file medinform_v13i1e77603_app1.docx]
