# Supplementary material for: Attitudes Toward Common Data Models Among Chinese Biomedical Professionals: Cross-Sectional Survey
Source: JMIR Med Inform. 2025 Nov 5;13:e77603. doi: 10.2196/77603 (PMC12631093; doi:10.2196/77603)
Supplement: Multimedia Appendix 2 [file medinform_v13i1e77603_app2.docx]

| 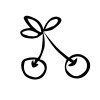 | **Checklist for Reporting Results of Internet E-Surveys (CHERRIES)** | |
| --- | --- | --- |
| ***Item Category*** | ***Checklist Item*** | ***Reporting Status*** |
| **Design** | Describe survey design | The target population was Chinese professionals in the medical field. The methods section explicitly states that a convenience sampling method was employed. |
| **IRB (Institutional Review Board) approval and informed consent process** | IRB approval | The ethical approval number (IRB00001052-24052) is provided in the dedicated 'Ethical approval' section. |
|  | Informed consent | We had a detailed informed consent before the questionnaire started, informing the participants that we would use their questionnaire results for scientific research, and the questionnaire would only begin when the participants clicked the consent button. |
|  | Data protection | Sojump safeguards user privacy through ​anonymous collection​ and ​technical isolation​ mechanisms. By default, questionnaire designers ​cannot directly access​ respondents' personally identifiable information, such as names and mobile phone numbers, as this data is ​anonymized and masked.  Furthermore, the platform employs robust security measures, including ​enterprise-grade firewalls, ​SSL encryption​ for data in transit, and encryption for data at rest. These measures are implemented to ​mitigate external attacks​ and prevent data breaches, thereby ensuring the overall ​security and integrity​ of all collected data. |
| **Development and pre-testing** | Development and testing | The survey was developed and refined through an iterative testing process. It was initially distributed via WeChat and evaluated by peers with research experience for clarity, flow, and technical functionality. The questionnaire was also reviewed by three associate professors, whose feedback informed revisions to its design, structure, and coherence. The updated version was retested to ensure all components worked correctly before public release. |
| **Recruitment process and description of the sample having access to the questionnaire**​ | Open survey versus closed survey | The survey was disseminated via WeChat, making it an open survey accessible to anyone with the link, without password protection. |
|  | Contact mode | The initial contact and recruitment of participants were conducted entirely online through the WeChat platform. |
|  | Advertising the survey | The survey was advertised and disseminated through the WeChat platform. |
| **Survey administration​** | Web/E-mail | The e-survey was web-based and hosted on the Sojump platform. It was distributed to participants through WeChat. Responses were captured automatically by the platform's system and did not require manual data entry. |
|  | Context | The questionnaire was distributed via WeChat, including within several groups consisting of medical field experts. |
|  | Mandatory/voluntary | Participation in the survey was voluntary. |
|  | Incentives | Our study did not report offering any incentives to participants. |
|  | Time/Date | Data were collected from September 18, 2024, to November 8, 2024. This timeframe was clearly stated. |
|  | Randomization of items or questionnaires | Although we acknowledge the value of randomizing items to reduce bias, we maintained a fixed order to preserve the logical flow and progressive structure of the questionnaire, where questions build upon each other from general to specific. |
|  | Adaptive questioning | Yes, adaptive questioning was implemented in the survey to reduce its overall length and complexity. Certain items were conditionally displayed based on respondents' previous answers. |
|  | Number of Items | Adaptive questioning was employed to streamline the survey. Initially, 10 items were displayed. Based on responses to specific items, additional questions were conditionally triggered, with the full set of 20 items presented on a single scrollable page.​ |
|  | Number of screens(pages) | A single scrollable page.​ |
|  | Completeness check | All items were marked as mandatory within the survey platform, preventing submission until every question had been answered.​ |
|  | Review step | The survey platform did not allow respondents to review or change their answers after moving to the next section.​ |
| **Response rates**​ | Unique site visitor | The questionnaire cannot collect IP addresses or cookies, since they are relatively private. Therefore, it is impossible to determine a unique visitor. |
|  | View rate (Ratio of unique survey visitors/unique site visitors) | Due to the privacy settings, which prevent the collection of IP addresses or cookies, it was not possible to determine or filter unique visitor. |
|  | Participation rate (Ratio of unique visitors who agreed to participate/unique first survey page visitors) | We cannot determine how many visitors visit the first page of the survey. |
|  | Completion rate (Ratio of users who finished the survey/users who agreed to participate) | Due to the technical enforcement preventing incomplete submissions, the completion rate for those who started the survey was effectively 100%. |
| **Preventing multiple entries from the same individual**​ | Cookies used | Although technical identifiers like cookies were not collected, the survey platform was linked to the WeChat authentication system. This integration ensured that each WeChat account could only submit the questionnaire once, effectively preventing duplicate entries from a single user. |
|  | IP check | Although technical identifiers like IP addresses were not collected, the survey platform was linked to the WeChat authentication system. This integration ensured that each WeChat account could only submit the questionnaire once, effectively preventing duplicate entries from a single user. |
|  | Log file analysis | We did not use other techniques to analyze the log file for the identification of multiple entries. |
|  | Registration | The survey platform was linked to the WeChat authentication system. This integration ensured that each WeChat account could only submit the questionnaire once, effectively preventing duplicate entries from a single user. |
| **Analysis**​ | Handling of incomplete questionnaires | As incomplete submissions were technically prevented, all analyzed questionnaires were complete. |
|  | Questionnaires submitted with an atypical timestamp | The median completion time was reported (121.5 seconds). All completion times were deemed valid as they satisfied the criterion of >2 seconds per question, and no exclusions based on timestamp were applied. |
|  | Statistical correction | No statistical correction methods (e.g., weighting, propensity scores) were employed to adjust for the non-representative sample, as the study was primarily descriptive. |
